# Consistency is key: sleep regularity predicts all-cause mortality

**DOI:** 10.1093/sleep/zsad285

**Published:** 2023-11-04

**Authors:** Faris M Zuraikat, Brooke Aggarwal, Sanja Jelic, Marie-Pierre St-Onge

**Affiliations:** Department of Medicine, Columbia University Irving Medical Center, New York, NY, USA; Department of Medicine, Columbia University Irving Medical Center, New York, NY, USA; Department of Medicine, Columbia University Irving Medical Center, New York, NY, USA; Department of Medicine, Columbia University Irving Medical Center, New York, NY, USA

Across many domains of life, consistency is considered beneficial, and sleep is no exception to this rule. Epidemiological evidence suggests that greater within-person consistency in sleep across nights relates to better health and performance [1], and irregularity in sleep–wake patterns has emerged as a prominent risk factor for incident cardiometabolic diseases [2–4] and cardiovascular events [5]. However, few studies have assessed prospective associations of within-person consistency in sleep–wake patterns, hereafter referred to as sleep regularity, with all-cause and cause-specific mortality. Furthermore, no prior study has compared directly the robustness of mortality risk prediction of sleep regularity versus other important dimensions of sleep, such as sleep duration.

In this issue of *SLEEP*, Windred et al. examined the role of sleep regularity in risk for mortality [6]. Using data from participants in the UK biobank who had completed 7 days of actigraphy during visits conducted between 2013 and 2016, the authors computed a sleep regularity index (SRI) score. Briefly, the SRI provides a granular measure of concordance in patterns of sleep and wake states across days of recording, with higher scores indicative of greater interdaily consistency in sleep–wake timing [6, 7]. Among the 60 997 middle-aged to elderly men and women with valid SRI data, 1859 deaths occurred between 2013 and 2021, equating to 4.84 deaths per 1000 person-years. The authors found that greater sleep regularity was associated with significantly lower risk for all-cause mortality. In fact, relative to the lowest quintile of SRI, the highest quintile, representing those with the most stable interdaily sleep–wake patterns, had 30% lower risk of mortality from all causes and 38% lower risk of cardiometabolic mortality. Interestingly, while sleep duration was also found to predict mortality incidence, SRI produced larger minimum risk estimates for each mortality outcome aside from cardiometabolic deaths. This pattern was confirmed in models directly evaluating the predictive value of SRI versus sleep duration. Most notably, SRI produced a better fit for all-cause mortality data than did sleep duration [6]. Thus, this study not only found that sleep regularity was a significant predictor of all-cause and cause-specific mortality but also suggests that consistency across nights in sleep–wake timing may be more strongly related to mortality risk than sleep duration.

Findings from Windred et al. [6] of an inverse relationship between sleep regularity and mortality risk have high public health relevance and support the need for this dimension of sleep to be considered in strategies for health promotion. For example, sleep duration has been formally recognized as a key determinant of cardiometabolic health [8], leading to its recent inclusion as one of the American Heart Association’s Essential eight components of cardiovascular health [9]. The study from Windred et al. [6] adds to a growing body of evidence that *regularity* of nightly sleep is an independent correlate of future morbidity and mortality that confers equal, if not greater, predictive value than sleep duration. While there is unequivocal evidence for a critical role of sleep duration in cardiometabolic health [8], irregular sleep, also a directly modifiable lifestyle behavior, is now strongly linked with both cardiometabolic costs [2] and mortality [6]. This highlights the need to address multiple sleep behaviors to reap the greatest cardiometabolic health benefits.

The study by Windred et al. also highlights areas for future research. As noted by the authors, there is a lack of diversity among UK Biobank participants, bringing into question the broader generalizability of findings. Therefore, it will be of particular importance to replicate these findings among racially and ethnically diverse samples given established sleep health disparities and their emerging contribution to broader health inequities [10]. Another question stemming from this investigation is whether SRI is the most useful tool to capture interdaily regularity in sleep patterns. While the SRI provides a unique, valid, and reliable measure of sleep regularity that confers predictive value for health outcomes, the means of deriving this measure is complex and requires granular data on sleep and activity. In contrast, other measures of sleep variability, such as the intra-individual standard deviation (SD) of sleep onset timing, can be computed more easily. Interestingly, the SRI appears to correlate with such measures in this sample of UK Biobank participants, as higher SRI scores corresponded with lower SD of sleep onset timing [6]. More studies are needed to compare the various methods of quantifying sleep regularity and identify a simple metric that could be used in helping people keep their sleep schedule more constant.

Maybe the most critical next step in this line of research will be to elucidate a causal pathway linking sleep regularity with health. In a landmark study of sleep regularity and cardiometabolic disease risk, Huang and Redline postulated that within-person deviations in the duration and/or timing of sleep likely represent the most prevalent cause of circadian disruptions at the population level [3]. That study catalyzed a surge of observational research evaluating relationships between sleep regularity and a broad range of cardiovascular and metabolic outcomes in various population cohorts. In reviewing these studies, we found consistent evidence that greater irregularity in sleep was associated with poorer cardiometabolic outcomes; this relationship was observed across studies in different populations and using different methods of quantifying sleep regularity (e.g. SD of sleep onset timing, SRI, and social jetlag) [2, 11]. Despite evidence for a significant role of sleep regularity in health we also noted an absence of research designed to evaluate a *causal impact* of sleep regularity on health outcomes [2].

To the best of our knowledge, no study has directly assessed the causal impact on health outcomes of experimental changes in sleep regularity. Indirect evidence was provided by a secondary analysis of data from the control condition of a separate trial, whereby healthy women who reduced their bedtime SD (i.e. stabilized nightly sleep) over 6 weeks, while maintaining normal sleep duration of 7–9 hours/night, had reductions in adiposity and markers of endothelial cell dysfunction relative to those who did not stabilize their sleep [12]. However, the exploratory nature of that analysis in a small group of women precludes conclusions about causal effects of improving sleep regularity on health outcomes. Thus, well-controlled randomized trials are needed to establish a causal relationship between sleep regularity and cardiovascular, endocrine, and metabolic outcomes and, importantly, to elucidate the pathways linking sleep regularity with key biological, physiological, and behavioral outcomes known to impact longer-term health. Such studies would complement the important epidemiologic findings of the present study [6] and, together, will be key to optimizing sleep health recommendations and interventions to promote health and longevity.
